# Pustular Psoriasis: From Pathophysiology to Treatment

**DOI:** 10.3390/biomedicines9121746

**Published:** 2021-11-23

**Authors:** Giovanni Genovese, Chiara Moltrasio, Nicoletta Cassano, Carlo Alberto Maronese, Gino Antonio Vena, Angelo Valerio Marzano

**Affiliations:** 1 Dermatology Unit, Fondazione IRCCS Ca’ Granda, Ospedale Maggiore Policlinico, 20122 Milan, Italy; giov.genov@gmail.com (G.G.); chiara.moltrasio@policlinico.mi.it (C.M.); carlo.maronese@unimi.it (C.A.M.); 2 Department of Pathophysiology and Transplantation, Università Degli Studi di Milano, 20122 Milan, Italy; 3 Department of Medical Surgical and Health Sciences, University of Trieste, 34137 Trieste, Italy; 4 Dermatology and Venereology Private Practice, 76121 Barletta, Italy; nicoletta.cassano@yahoo.com (N.C.); ginovena@gmail.com (G.A.V.)

**Keywords:** pustular psoriasis, generalized pustular psoriasis, palmoplantar pustular psoriasis, palmoplantar pustulosis, impetigo herpetiformis, acrodermatitis continua of Hallopeau, clinical features, pathogenesis, therapy

## Abstract

Pustular psoriasis (PP) is a clinicopathological entity encompassing different variants, i.e., acute generalized PP (GPP), PP of pregnancy (impetigo herpetiformis), annular (and circinate) PP, infantile/juvenile PP, palmoplantar PP/palmoplantar pustulosis, and acrodermatitis continua of Hallopeau (ACH), which have in common an eruption of superficial sterile pustules on an erythematous base. Unlike psoriasis vulgaris, in which a key role is played by the adaptive immune system and interleukin (IL)-17/IL-23 axis, PP seems to be characterized by an intense inflammatory response resulting from innate immunity hyperactivation, with prominent involvement of the IL-36 axis. Some nosological aspects of PP are still controversial and debated. Moreover, owing to the rarity and heterogeneity of PP forms, data on prognosis and therapeutic management are limited. Recent progresses in the identification of genetic mutations and immunological mechanisms have promoted a better understanding of PP pathogenesis and might have important consequences on diagnostic refinement and treatment. In this narrative review, current findings in the pathogenesis, classification, clinical features, and therapeutic management of PP are briefly discussed.

## 1. Introduction

Pustular psoriasis (PP) encompasses a heterogeneous group of nosological entities sharing cardinal clinicopathological features [1]. Such diseases are typified by an eruption of superficial sterile pustules, usually on an erythematous base. Histopathological features include hyperkeratosis, parakeratosis, acanthosis, diffuse dermal mononuclear and neutrophilic inflammatory infiltrates, intraepidermal collections of neutrophils, rete ridge elongation and dilated tortuous vessels in the papillary dermis [2,3].

The relative rarity and heterogeneity of PP forms have hampered the collection of precise data on prognosis and management. Recent progresses in the identification of genetic mutations and immunological mechanisms have led to a better understanding of PP pathogenesis and might have relevant consequences on diagnostic refinement and treatment.

## 2. Nosological Aspects

Some nosological aspects of PP are still debated. The classification of PP distinguishes localized variants [palmoplantar PP (PPPP) and acrodermatitis continua of Hallopeau (ACH)], and generalized variants, i.e., acute generalized PP (GPP), PP of pregnancy [also known as impetigo herpetiformis (IH)], annular (and circinate) and infantile/juvenile PP [1,4]. Localized forms of annular PP also exist [4].

In the consensus statement published by the European Rare and Severe PsO Expert Network (ERASPEN), PP was classified into three distinct types: (1) GPP, either relapsing (more than one episode) or persistent (more than 3 months), that can occur with or without psoriasis vulgaris (PV) and with or without systemic inflammation; (2) palmoplantar pustulosis (PPP); (3) ACH [5]. PPP and ACH are characterized by persistent pustules affecting palmoplantar surfaces and nail apparatus, respectively, with or without PV.

GPP is a rare, unpredictable, potentially life-threatening disease, with an acute, subacute, or more rarely chronic presentation and a variable clinical severity [1]. Typical forms of GPP are characterized by intermittent flares that can have a self-remitting course and can be interspersed with periods of partial or complete remission [6]. Acute GPP, the von Zumbusch variant, has an abrupt onset associated with systemic symptoms.

The appropriate classification of PPP and its inclusion within the spectrum of PP are still controversial. Japanese authors tend to consider PPP as a distinct nosological entity, in contrast with others, mainly outside of Japan, who regard PPP as synonymous with PPPP [4,6,7,8]. Emerging differences in genetic background, comorbidities and precipitating factors seem to favor the first hypothesis in the opinion of some authors [4]. PPP is sometimes referred to as PPPP in the presence of concomitant PV and/or a family history of PV [1,9,10]. A transcriptome analysis was performed in patients diagnosed clinically as having PPPP and PPP (distinguished on the basis of the presence or absence of PV lesions outside palms and soles and history of PV, respectively) [11]. Lesional skin gene expression appeared to be similar in PPP and PPPP, suggesting that they are not distinct clinical entities.

### 2.1. Clinical Variants

PP includes different, rare clinical variants that can occur either concurrently with or independently from PV. Coexistence of multiple variants (mixed forms) is possible [5,12] In general, PP is more common in women and more prevalent among Asians [1].

Mean age at onset seems to differ across disease types. A study in 863 patients with different PP forms belonging to different ethnic groups, predominantly European and Asian, demonstrated that the mean age at onset was lower in patients with GPP (31.0 ± 19.7 years) than in those with PPP (43.7 ± 14.4 years) or ACH (51.8 ± 20.4 years) [12].

#### 2.1.1. Von Zumbusch Psoriasis (Acute Generalized Pustular Psoriasis)

Von Zumbusch psoriasis is the most severe form of GPP. It is characterized by an acute, widespread eruption of multiple sterile pustules over the body, associated with extracutaneous symptoms, such as fever, chills, fatigue, nausea, and anorexia [13,14]. Erythroderma may occur [14]. Rarer mucocutaneous manifestations include subungual pustules, uveitis, cheilitis and geographic tongue [13,14,15]. The most common laboratory abnormalities are leucocytosis with neutrophilia and elevated inflammatory markers, but liver test abnormalities, hypocalcemia, and hypoalbuminemia can also be present [6,14,15]. Polyarthralgias and neutrophilic cholangitis have also been described [16,17], as well as a high frequency of thyroid dysfunction [18]. Previous or concurrent PV was documented in approximately half of acute GPP patients [12,15,16,19]. Nevertheless, GPP and PV appear to be genetically and mechanistically distinct [20].

An important differential diagnosis of acute GPP is acute generalized exanthematous pustulosis (AGEP) (Table 1), that may be difficult clinically and histologically [2,21,22,23,24]. Interestingly, AGEP and GPP also share genetic and immunological features [25,26,27,28].

Von Zumbusch psoriasis may exhibit an unfavourable prognosis, especially in cases evolving from ACH [1,29]. Mortality in GPP has mostly been attributed to complications resulting from sepsis, acute respiratory distress syndrome and cardiac failure [14]. Secondary amyloidosis is mentioned as another potential complication of GPP [30].

#### 2.1.2. Impetigo Herpetiformis (Pustular Psoriasis of Pregnancy)

IH is a form of pregnancy-associated PP first described in 1872 by Ferdinand von Hebra [31]. It is controversial whether this entity should be considered distinct from PP [32,33]. It is most frequently observed in the last trimester of pregnancy [34]. Multiple sterile pustules on an erythematous base are observed in an annular configuration at the folds with subsequent spreading over the body [34,35]. Systemic symptoms, such as fever, chills, fatigue, nausea, diarrhea, and polyarthralgias, may be observed [32,33,34]. Leukocytosis with neutrophilic predominance, anemia, increased erythrocyte sedimentation rate, hypocalcemia, hypophosphatemia and hypoalbuminemia are notable laboratory findings [33,35]. IH, especially if severe and long-lasting, may lead to poor neonatal outcomes, including placental insufficiency, fetal abnormalities, stillbirth, and early neonatal death [36], as well as to maternal death [31]. Most patients experience prompt remission in the post-partum period [37,38]. Recurrences with subsequent pregnancies are frequent, sometimes with earlier onset and greater severity, and can also occur while on treatment with oral contraceptives [33,39,40].

#### 2.1.3. Annular Pustular Psoriasis

Circinate or annular PP is a rare variant, consisting in an erythematous eruption with annular or polycyclic lesional morphology, small sterile pustules at the periphery of the lesions, and fine desquamation with mild-to-no systemic symptoms. Usual sites of involvement are flexural areas of the trunk and proximal extremities, buttocks and abdomen [41]. Formerly included within the spectrum of GPP, it is now considered a separate entity [42], with a preference for younger age groups and an overall benign, sub-acute and self-resolving course.

#### 2.1.4. Infantile/Juvenile Pustular Psoriasis

PP is extremely rare in patients aged under 18 years [43], with evidence limited to individual reports and small case series [44,45,46]. Onset of infantile/juvenile PP typically occurs at 6 to 7 years [47]. The clinical presentation is either with a circinate/annular lesional pattern, which is more common, or GPP-like [48]. Systemic involvement with fever, leucocytosis and elevation of acute phase reactants is possible [45].

#### 2.1.5. Palmoplantar Pustulosis/Palmoplantar Pustular Psoriasis

PPP involves the palms and/or soles exclusively and presents with sterile pustules, background erythema, hyperkeratosis and scaling [5,49]. Cutaneous involvement favors the thenar, hypothenar and central areas of the palms, and the soles at the corresponding levels [8,50]. Coalescence of pustules and hyperpigmentation after resolution are common features. PPP has been differentiated into two types by some Japanese authors: type A, as originally reported by Andrews, in which vesicles precede pustules and the association with PV is rare, and type B, as reported by Barber, characterized by a frequent association with PV and by the presence of pustules without a vesicular component [51]. PPP is regarded as a common dermatosis in Japan where its classical presentation is consistent with Andrews’ type A-PPP [51]. PPP typically affects middle aged women, especially smokers [8,13,50]. Possible comorbidities are arthro-osteitis, metabolic syndrome and thyroid disease [4,10,50]. The Japanese literature reports that around 30% of PPP patients develop musculoskeletal manifestations [9]. Finally, PPP is a frequent feature of the so-called SAPHO syndrome [9].

#### 2.1.6. Acrodermatitis Continua of Hallopeau

ACH is a very rare, localized form of PP with a chronic course and characteristic involvement of the distal digits and nail apparatus [52,53]. It is more common in middle-aged females.

ACH can progress to GPP, including severe acute GPP, strengthening the concept of a shared disease spectrum [52,53,54]. ACH is characterized by painful sterile pustules on the distal portions of the fingers and toes with severe nail involvement, possibly leading to onychodystrophy and anonychia. Osteitis can occur, rarely resulting in osteolysis of the distal phalanges [52,53,55]. 

Its differential diagnosis includes other acral pustular conditions, including PPP and infectious dermatoses. Localization in periungual areas and a tendency to remain localized to a limited number of digits for many years are useful diagnostic clues [56,57].

## 3. Pathogenesis

Despite some degree of overlap, clinical, histological, and pathophysiological differences exist between PP and PV.

The available data seem to indicate that PP is dominated by an intense inflammatory response resulting from innate immunity hyperactivation, with a crucial involvement of the interleukin (IL)-36 axis, while a prominent role of the adaptive immune system and IL-17/IL-23 axis is seen in PV.

IL-36 cytokines belong to the IL-1 superfamily and comprise three proinflammatory agonists, IL-36α, -β, -γ, and one receptor antagonist (IL-36Ra). IL-36 cytokines are expressed in various cell types, including keratinocytes and immune cells [58,59], and are abundantly present in the skin [60]. They are released as a precursor and require processing by specific proteases, especially derived from neutrophils, to become bioactive [61,62,63,64]. IL-36α, -β, and -γ through the IL-36 receptor (IL-36R) activate nuclear factor-κB (NF-κB) and mitogen activated protein kinase (MAPK), thus inducing the activation of downstream pathways responsible for the production of pro-inflammatory cytokines, chemokines and costimulatory molecules. IL-36Ra acts as regulator, by competing with the agonistic IL-36 cytokines for attachment to IL-36R [65,66]. 

IL-36 agonists also signal to keratinocytes in an autocrine manner and regulate T cell proliferation and polarization and dendritic cell maturation [64,65,66,67], supporting a possible involvement in the crosstalk between innate and adaptive immunity [65]. IL-36 may stimulate IL-17 pathway either directly or through IL-23 induction and synergize with IL-17A [68].

A number of allelic variants have been found to cause or contribute to PP onset or susceptibility, primarily GPP [1,69]. The interaction between genetic predisposition and environmental factors is thought to have a relevant pathogenetic role. Stress, infections, pregnancy and rapid withdrawal of systemic corticosteroids have been hypothesized to precipitate and/or trigger various PP forms [15,70,71,72,73]. Vaccination has been identified as a possible precipitating factor particularly in juvenile forms [74]. Recently, PP has been described in the setting of Coronavirus disease 2019 (COVID-19) [75], as well as after COVID-19 vaccination [76]. Hypocalcemia is mentioned as a potential trigger, particularly in IH, even if evidence supporting causality is lacking [34,77,78]. 

Different medications have also been implicated, including terbinafine, penicillin, lithium, iodine, progesterone, hydroxychloroquine, some nonsteroidal antinflammatory drugs, topical irritating agents and even anti-tumor necrosis factor (TNF) agents and more rarely other biologics [1,79,80,81]. Treatment with TNF-α inhibitors can cause paradoxical flares of PP, especially PPPP [82,83], but the underlying pathomechanism remains to be elucidated.

The main triggering factors of PPP include smoking, metal hypersensitivity, focal infections (e.g., tonsillitis, chronic sinusitis, and dental infections), stress, and drugs [84,85,86,87]. Precipitating factors most closely associated with ACH are localized trauma to the distal portion of a digit and localized infections [13,88,89].

### 3.1. Pathophysiology of Generalized Forms

Immunological pathways in GPP and PV are thought to be partly overlapping, with a more prominent role of the innate immune system, IL-1 and IL-36 in GPP pathogenesis. However, TNF-α and IL-17A also seem to be involved [62,90]. A gene expression study on lesional skin revealed a significant overexpression of IL-17A, TNF, IL-1, IL-36 and interferons in both PV and GPP with a significantly greater abundance of transcripts for IL-1β, IL-36α and -γ in GPP versus PV lesions [62].

The pathogenic landscape of IH is poorly understood [32,91,92] and a key issue is the relationship with GPP. During pregnancy, immune responses shift towards an overall Th2 polarization, although emerging evidence indicates a much more nuanced immunological rewiring. Recent research hints at a regulatory role for IL-36 cytokines in the immunology of reproduction [93] providing a conceptual framework for the understanding of PP during pregnancy, which could represent the result of deranged IL-36 cytokines physiologic changes in the setting of a predisposing genetic background.

Annular PP-like lesions have been reported in few cases of hereditary lactate dehydrogenase M-subunit deficiency, that is responsible for an imbalance between oxidized and reduced forms of nicotinamide adenine dinucleotide (NAD/NADH), leading to dysregulation of intracellular calcium. Takeo et al. hypothesized that calcium dysregulation may lead to epidermal infiltration by neutrophils and elevated serum levels of IL-8, two hallmarks of PP [94]. Annular PP was observed in an individual with Noonan syndrome, supporting a putative shared pathomechanism involving the RAS/MAPK signaling pathway [95].

As concerns genetic factors in GPP, a list of the main genes involved in GPP pathogenesis is summarized in Table 2 [1,69,96,97,98,99,100].

Loss-of-function pathogenic variants of the *IL36RN* gene, encoding IL-36Ra, have been found with a frequency close to 24% in GPP [1,12]. More specifically, mutations in *IL36RN* have been shown to be associated with GPP without PV and with an earlier age of disease onset [69,101,102].

Deficiency of IL-36Ra (DITRA) has been classified as a subgroup of GPP with a specific monogenetic defect [103] consisting in null mutations of *IL36RN*, associated with severe clinical phenotypes. Hypomorphic variants with decreased or unchanged protein expression have also been found and may account for clinical heterogeneity of GPP [104].

*IL36RN* variants have been detected also in IH, and, in particular, East Asian founder mutations might be implicated in IH pathogenesis [105]. Curiously, identical *IL36RN* mutations led to both isolated IH and IH with a preceding history of GPP and/or PV.

Recently, *CARD14* causal variants have been linked to GGP. Heterozygous gain-of-function variants in *CARD14* occur in up to 21% of GPP patients with concomitant PV [1,97] while a homozygous gain-of-function *CARD14* variant has been described in a mild case of IH [106].

Accordingly, *IL36RN*-related pustulosis and *CARD14*-mediated PP have been classified within the spectrum of autoinflammatory keratinization diseases, a group of inflammatory keratinization disorders with autoinflammatory pathomechanisms [107].

*AP1S3* is another gene recently associated with GPP [108]. *AP1S3* pathogenic variants are mainly found in Europeans and rarely in East Asians [69]. *AP1S3* mutations can be co-inherited with *IL36RN* genetic changes, modifying the phenotypic effect of the latter [98].

The mutational analysis of *IL36RN, CARD14,* and *AP1S3* genes in a group of 61 GPP patients showed that almost two-thirds of them did not carry variants in any of the three genes, reiterating the complexity of GPP pathogenesis [102].

Additional genetic factors might contribute to GPP pathogenesis (Table 2), like *SERPINA3* [109], *TNIP1* [110,111], and *MPO* [63,112] variants. *MPO* gene variants can increase neutrophil counts and activity of neutrophil serine proteases, capable of activating IL-36 precursors [63,69]. Interestingly, GPP was also documented in two patients with mycobacterial infection and interferon (IFN)-γ receptor deficiency, due to *IFNGR1* or *IFNGR2* gene mutations [113].

### 3.2. Pathophysiology of Localized Forms

Many authors in support of the theory that PPP is a distinct entity from palmoplantar PV argue genetic differences [4,20,51,114,115].

*IL36RN* pathogenic variants have been revealed in approximately 5% of PPP patients [12], and hypomorphic variants seem to be relatively more prevalent in localized forms of PP as compared to GPP [6]. Further, in a small number of PPP patients, also *APS13* and *CARD14* pathogenic variants have been detected [108,116]. Although the pathophysiology of PPP remains obscure, it is now widely accepted that the role of the eccrine sweat gland is critical. Specifically, the acrosyringium serves as the primary site of inflammation and pustule formation [51,117]. Interestingly, an increase in Langerhans cells can be found in both lesional and non-lesional skin of PPP patients, indicating an antigen-driven process [118]. The antimicrobial peptide hCAP-18/LL37 appears to act as an inducer of inflammation in PPP by upregulating the levels of pro-inflammatory cytokines [119].

IL-17A is highly expressed in the palms and soles of PPP patients in comparison to healthy subjects, while IL-12 and IL-23 are not predominant [117]. 

The association between smoking and PPP is well recognized [50,84,120]. Smoking has been demonstrated to increase IL-17 levels [117,121]. Moreover, the expression of the acetylcholine receptor α7nAChR within the acrosyringium may be decreased by smoking, with consequent impairment of the activation of the endogenous nicotinic anti-inflammatory pathway [122]. Conversely, smoking cessation can lead to improvement of PPP [123].

Likewise, the pathophysiology of ACH has long been debated. Case reports documenting ACH transitioning to GPP together with the possible association with *IL36RN*, *CARD14* and *AP1S3* variants support the existence of shared disease spectrum, with ACH at one end and GPP at the other [12,52,53,54].

## 4. Treatment

Therapeutic management of PP is challenging and depends on multiple factors [124,125], especially disease severity, extent of involvement and patients’ comorbidities. Owing to the rarity of PP, clinical trials focused on PP forms are scarce and evidence-based guidelines for treatment are lacking [124].

Table 3 contains information about the main recent clinical trials regarding treatment of PP [126,127,128,129,130,131,132,133,134,135,136,137,138,139,140,141,142,143,144,145,146,147,148,149,150]. Ongoing clinical trials and other completed studies not included in Table 3 are reported in Table 4.

### 4.1. Treatment of Generalized Pustular Psoriasis

Acitretin, cyclosporine, methotrexate and infliximab have been indicated as first-line therapies for GPP [125,151]. Retinoids are considered one of the preferred treatment options [125,152]. Due to their quick onset of action, infliximab or cyclosporine may be useful in severe and extensive disease [125,153]. According to US Medical Board of the National Psoriasis Foundation recommendations published in 2012 [125], second-line treatments are adalimumab, etanercept, psoralen plus ultraviolet-A (PUVA) phototherapy, topical therapy (corticosteroids, calcipotriol, and tacrolimus, for more localized disease or as adjunctive tools) or combination therapy for recalcitrant disease, which can comprise a first-line systemic conventional agent associated with a biologic drug such as an anti-TNF agent [125].

Dapsone has also been proposed as a possible therapeutic option [30,154]. 

Systemic corticosteroids are usually discouraged–with a few exceptions–due to the increased risk of pustulation, as well as flares during treatment or upon discontinuation [30,155], although a recent study has shown low rates of psoriasis flare in such circumstances [156].

Weighing in on the relative efficacy of available biologics in GPP, TNF-α inhibitors, in particular infliximab, and ustekinumab, an anti-IL-12/23 monoclonal antibody, seem to be backed up by more robust evidence [153,157]. Additional biologic therapies have been evaluated. The IL-1 receptor antagonist anakinra and IL-1β inhibitors have been successfully administered in isolated cases [158,159,160]. Most recently, the anti-IL17A monoclonal antibodies secukinumab and ixekizumab and the anti-IL-17A receptor monoclonal antibody brodalumab have shown encouraging results [130,133,134,135,161]. Guselkumab, an anti-IL-23p19 agent, demonstrated efficacy in Japanese patients with GPP [136], whereas a phase III trial with risankizumab, another IL23p19 inhibitor, in Japanese GPP patients has been completed (Table 4).

Noteworthily, there appears to be no influence of *IL36RN* mutational status on treatment outcome in GPP patients treated with biologics [162], but further studies are needed.

Interesting data have been collected from real-life experiences. An analysis of 1516 Japanese patients with GPP hospitalized from July 2010 to March 2019 showed better outcomes with biologics compared to other treatments, but patients treated with biologics were younger and had fewer comorbidities. IL-17 inhibitor use was associated with comparable in-hospital mortality and morbidity to those of TNF inhibitors. Indeed, about half of the patients in the biologics group were treated with concomitant oral agents, sometimes in addition to systemic corticosteroids [155].

In a retrospective German multicenter study examining 201 treatment series of 86 GPP patients, biological treatment was found to be significantly more effective than non-biological therapies and the median drug survival was significantly higher with biologicals vs. nonbiologicals. When the drugs were grouped according to the target cytokine, the best retention time was observed for IL-17A inhibitors, followed by IL-(12)/23 inhibitors and TNF-α blockers [163].

In a phase I proof-of-concept study in 7 patients with a GPP flare, a rapid improvement was obtained after a single intravenous dose of spesolimab, a novel anti-IL-36R antibody [126]. The drug proved to be effective regardless of the *IL36RN* mutational status and is currently being investigated in further trials [164,165] (Table 4).

Imsidolimab is another anti-IL36R monoclonal antibody currently under investigation for GPP (Table 4).

Although not widely available, granulocyte and monocyte adsorption apheresis (GMA) has yielded positive results in the management of GPP [30,166].

Limited data exist for pediatric PP. Among the conventional systemic drugs, oral retinoid treatment is the most commonly administered, even if there are concerns about growth disturbances. Cyclosporine and methotrexate have also been used as first-line treatment, whereas etanercept may be regarded as one of the preferred second-line choices for children with GPP [167].

### 4.2. Treatment of Pustular Psoriasis of Pregnancy

The risk of complications implies the need for close monitoring and adequate supportive treatment. Early induction of labor, if appropriate, is suggested in the management of severe or refractory IH [168]. The data regarding treatment of PP of pregnancy are extremely limited. Current treatment regimens include systemic corticosteroids, which are the most frequently used drugs, cyclosporine, narrow-band ultraviolet-B (NB-UVB), adjuvant antibiotic therapy and topical agents [30,36,78,91,125]. Biologic therapy can be cautiously considered for severe refractory IH, and there are only very few reports mostly regarding TNF inhibitors, especially infliximab [30,78,125]. Certolizumab might be an interesting therapeutic agent for IH in terms of safety for the mother and fetus [169]. GMA appears to be particularly appealing during pregnancy as it represents one of the safest therapeutic options [166].

### 4.3. Treatment of Palmoplantar Pustulosis/Palmoplantar Pustular Psoriasis

PPPP/PPP is notoriously treatment-refractory [170]. The most commonly used treatments remain topical agents, mainly topical corticosteroids regarded as more effective if used under occlusion [171]. Other topical therapies are vitamin D derivatives, topical PUVA, photodynamic therapy and tacrolimus [10,125]. Topical treatment is frequently not satisfactory and systemic treatment is therefore required [6].

First-line systemic treatments for PPPP are represented by cyclosporine, retinoids and oral PUVA or retinoid-PUVA [125]. Among systemic non-biological agents, cyclosporine has the highest level of evidence for efficacy in PPPP [6]. Nevertheless, high-quality evidence is lacking for most PPP treatments [171]. Other oral treatments include tetracyclines [171] and the phosphodiesterase inhibitor apremilast that has been described as effective in few patients with moderate-to-severe or refractory disease [10,146,172]. 

The successful use of TNF-α inhibitors or ustekinumab in PPP/PPPP has been documented in case reports and small studies [10,49]. However, a randomized placebo-controlled trial failed to demonstrate a statistically significant efficacy of ustekinumab in PPPP and PPP patients [117].

Treatment with brodalumab was unsuccessful or only moderately effective in a series of 4 PPPP patients [173].

Data from the 2PRECISE trial showed that at week 52 the Palmoplantar Psoriasis Area and Severity Index (PPPASI) had at least a 75% reduction from baseline (PPPASI-75) in 41.8% of subjects treated with 300 mg/month of secukinumab [131,132]. Despite potential benefits, the primary end point (PPPASI-75 response with secukinumab at week 16 versus placebo) was not met.

The therapeutic potential of guselkumab was revealed in Japanese patients with moderate-to-severe PPP. A significantly higher proportion of patients in the guselkumab 100-mg group achieved at least 50% reduction of PPPASI (PPPASI-50) at week 16 versus placebo, but the result was not significant for the guselkumab 200-mg group [138].

In a phase 2a study investigating the efficacy of spesolimab in PPP, the primary endpoint (PPPASI-50 at week 16) was not met, although post hoc analyses demonstrated a greater efficacy of spesolimab over placebo in patients with more severe disease [127].

Imsidolimab was shown not to determine a significant improvement over placebo in a phase 2 clinical trial in moderate-to-severe PPP [174].

### 4.4. Treatment of Acrodermatitis Continua of Hallopeau

Treatment options for ACH, which is particularly treatment-refractory, are mainly grounded on data from case reports. Several therapeutic options have been tried with variable and sometimes equivocal results [53,55]. Topical treatments (i.e., corticosteroids, calcipotriol, tacrolimus, and fluorouracil, or a combination of these drugs) have a limited efficacy and alternative treatments are often necessary. These include cyclosporine, systemic corticosteroids, retinoids, methotrexate, PUVA, UVB phototherapy, GMA, and biologic agents (e.g., anti-TNF agents, IL-17 inhibitors, IL-12/23 inhibitors, and anakinra) [53,55,170] and also apremilast and baricitinib [175,176]. 

In a series of 39 patients with ACH, the overall effectiveness of systemic treatments was low (excellent response rate: 14.8%) [177]. A treatment algorithm was suggested, starting with acitretin or methotrexate as first-line therapy, followed by biologics, particularly adalimumab and secukinumab, and possibly guselkumab, whereas cyclosporin might be used for short-term control [177].

## Figures and Tables

**Table 1:** Main clinical characteristics of acute generalized exanthematous pustulosis (AGEP) and differentiation from acute generalized pustular psoriasis (GPP).

Main features of AGEP	Rare disorder attributed mostly to drugs (infections, hypersensitivity to mercury and spider bite have sporadically been implicated) Sudden occurrence of a generalized skin rash with sterile nonfollicular pinhead-sized pustules on an oedematous erythema, often associated with systemic symptoms, including fever, leucocytosis and neutrophilia Spontaneous resolution of pustules within a few days followed by pin-point desquamation Mild, nonerosive mucous membrane involvement (mostly oral) in about 20% of cases
Factors favoring the diagnosis of AGEP over GPP	Absence of family/personal history of psoriasis (however, history of psoriasis possible in AGEP) Recent drug administration (very frequent in AGEP, possible but less frequent in GPP, that can also be drug-elicited) Predominance of lesions in the folds, especially at the onset Shorter duration of fever and pustules Spontaneous rapid resolution (within 15 days after withdrawal of the culprit drugs) and nonrecurrent tendency Arthritis (rare in AGEP, affecting about 30% of cases in GPP) Peripheral eosinophilia (present in about one-third of cases and usually mild) Histopathological features: - superficial spongiform pustules, exocytosis of neutrophils and eosinophils, occasional necrotic keratinocytes, papillary dermal oedema, mixed dermal infiltrates containing neutrophils and eosinophils (classical psoriasis changes infrequent and usually mild) - presence of eosinophils, absence of PDCs and absence of tortuous dilated capillaries favoring a diagnosis of AGEP over PP (perivascular and intraepidermal PDCs, dilated tortuous vessels and MxA expression in the dermal inflammatory infiltrate significantly in favor of PP)

Information extracted from References [2,21,22,23,24]. AGEP: acute generalized exanthematous pustulosis; GPP: generalized pustular psoriasis; MxA: human myxovirus resistance protein 1; PDCs: plasmacytoid dendritic cells; PP: pustular psoriasis.

**Table 2:** Genes involved in the pathogenesis of GPP with description of related proteins encoded and their functions.

Gene Involved	Protein Encoded	Main Functions of the Protein
*IL-36RN*	IL-36Ra	Inhibition of the proinflammatory effects of IL-36, competing with the agonistic IL-36 cytokines for the attachment to IL-36R
*CARD14*	CARD14 (CARMA2) Membrane-associated guanylate kinase scaffolding protein, predominantly expressed in keratinocytes	NF-kB and MAPK activation through the formation of a signaling complex with BCL10 and MALT1
*AP1S3*	σ1C subunit of the AP-1 complex	AP-1 complex is involved in clathrin-mediated vesicular trafficking between the trans-Golgi and the endosomes, autophagosome formation, Toll-like receptor homeostasis and keratinocyte autophagy
*MPO*	Myeloperoxidase Cationic heme-containing enzyme found in neutrophil azurophil granules	Essential to the antimicrobial activity of neutrophils, it is involved in reactive oxygen species production and phagocytosis, as well as in the generation of neutrophil extracellular traps
*SERPINA3*	Serpin A3 Serine protease inhibitor	Interaction with the neutrophil protease cathepsin G and other proteases to inhibit their activity
*TNIP1*	Tumor necrosis factor-alpha induced protein 3 interacting protein 1	Interaction with zinc finger protein A20 to inhibit NF-κB signalling (other targets include the RARs-α and -γ, and peroxisome proliferator-activated receptors)

Information extracted from References [1,69,96,97,98,99,100] AP-1: adaptor protein complex 1; AP1S3: adaptor related protein complex 1 subunit sigma 3; BCL10: B-cell lymphoma/leukemia 10; CARD14: caspase recruitment domain-containing protein 14; GPP: generalized pustular psoriasis; IL: interleukin; IL-36R: interleukin-36 receptor; IL-36Ra: interleukin-36 receptor antagonist; MALT1: mucosa-associated lymphoid tissue lymphoma translocation protein 1; MAPK: mitogen-associated protein kinase; NF-κB: nuclear factor-κB; RAR: retinoic acid receptor.

**Table 3:** Principal recent clinical trials regarding treatment of patients with GPP and PPP/PPPP.

Drug	References	Study Type (Randomization Ratio, If Applicable)	Identifier Number, If Applicable	Participants *	Details of Treatment (AT in Placebo-Controlled Studies)	Main Efficacy Results (Primary Outcome, If Applicable)
Spesolimab (anti-IL-36 receptor mAb)	[126]	Phase 1, proof-of-concept, OL, SA	ClinicalTrials.gov NCT02978690	7 patients with GPP flare	Single intravenous dose at 10 mg/kg	At week 4, GPPGA score of 0 or 1 (“clear” or “almost clear”) in all patients, and mean GPPASI improvement from baseline of 79.8%
	[127]	Phase 2a, DB, RPC (1:1:1)	ClinicalTrials.gov NCT03135548	59 PPP patients	900 mg or 300 mg intravenously every 4 weeks at Day 1, 29, 57 and 85	PPPASI50 response at week 16 in 31.6% in each of the two AT groups vs. 23.8% in the placebo group (N.S.)
Adalimumab (anti-TNF-alpha mAb)	[128]	Phase 3, OL, SA	ClinicalTrials.gov NCT02533375	10 Japanese GPP patients	80 mg s.c. at week 0 followed by 40 mg every other week: last dose at week 50 (escalation to 80 mg every other week at week 8 or later, if necessary)	Clinical response [remission (TSS 0) or improvement (reduction of ≥1 point from a baseline TSS of 3 or ≥2 points from a baseline TSS of ≥4)] at week 16 in 70% (*n* = 10)
Infliximab (anti-TNF-alpha mAb)	[129]	Phase 3, OL, SA SPREAD study	ClinicalTrials.gov NCT01680159	7 Japanese GPP patients with loss of efficacy to standard-dose maintenance therapy	Escalation to 10 mg/kg (intravenous infusion) every 8 weeks	Severity graded as mild in 71% and moderate in 29% at week 0, and mild in all patients at weeks 24 and 40
Secukinumab (anti-IL-17A mAb)	[130]	Phase 3, OL, SA	ClinicalTrials.gov NCT01952015	12 Japanese GPP patients	150 mg s.c. at week 0, 1, 2, 3 and 4, and then every 4 weeks until week 52 (300 mg in 2 non-responders)	At week 16, treatment success in 83.3% (*n* = 10) [CGI of “very much improved” (*n* = 9) or “much improved” (*n* = 1)]
	[131]	Phase 3b, DB, RPC (1:1:1) 2PRECISE study	ClinicalTrials.gov NCT02008890	237 patients with moderate-to-severe PPPP	300 mg or 150 mg s.c. at weeks 0, 1, 2, 3, and 4, and then every 4 weeks until week 52	At week 16, PPPASI75 response in 26.6% of patients with high-dose AT, 17.5% with low-dose AT and 14.1% of patients who received placebo (N.S.)
	[132]	Extension period for patients with meaningful clinical response after completion of the 2PRECISE study	ClinicalTrials.gov NCT02008890	94 PPPP patients in total	Extension of AT after week 52 up to 148 weeks	At week 148, PPPASI75 response rates increased in all groups, with similar levels for placebo/low-dose AT (75%), placebo/high-dose AT (77.8%), and initial high-dose AT (78.3%), and 100% responders in the initial low-dose AT group
Ixekizumab (anti-IL-17A mAb)	[133,134]	Phase 3, OL, SA UNCOVER-J study	ClinicalTrials.gov: NCT01624233	5 Japanese GPP patients	160 mg at week 0, 80 mg every 2 weeks from week 2 to week 12, 80 mg every 4 weeks thereafter up to week 244	GIS of “resolved” or “improved” in all patients from week 12 onward
Brodalumab (anti-IL-17 receptor A mAb)	[135]	Phase 3, OL, SA	ClinicalTrials.gov NCT01782937	12 Japanese GPP patients	140 mg s.c. at weeks 0, 1 and 2, and then every 2 weeks until week 52 (escalation to 210 mg at week 4 and beyond, if necessary)	CGI of “improved’ or ‘remission’ in 83.3% at week 12 and 91.7% at week 52
Guselkumab (anti-IL-23p19 mAb)	[136]	Phase 3, OL, SA	ClinicalTrials.gov NCT02343744	10 Japanese GPP patients (9 evaluable)	50 mg s.c. at weeks 0, 4 and every 8 weeks until week 52 (beginning at week 20, escalation to 100 mg every 8 weeks, if necessary)	At week 16, treatment success in 77.8% [CGI of “very much improved” in 2 patients, “much improved” in 2, and “minimally improved” in 3 subjects)
	[137]	Phase 2, proof-of-concept, DB, RPC (1:1)	ClinicalTrials.gov NCT01845987	49 Japanese PPP patients	200 mg s.c. at weeks 0 and 4	Reduction in mean PPSI total score from baseline at week 16 −3.3 in the AT group vs. −1.8 in the placebo group (difference in LS mean, −1.5; 95% CI, −2.9 to −0.2; *p* = 0.03)
	[138]	Phase 3, DB, RPC (1:1:1)	ClinicalTrials.gov NCT02641730	159 Japanese patients with refractory PPP	100 mg or 200 mg s.c. at weeks 0, 4, and 12, and every 8 weeks thereafter	At week 16, LS mean change in PPPASI score from baseline −15.3 (*p* < 0.001) for the low-dose AT group and −11.7 (*p* = 0.02) for high-dose AT group vs. −7.6 for the placebo group
	[139]	Extension period	ClinicalTrials.gov NCT02641730	133 patients completed the study at week 84	Treatment until week 60. At week 16, re-randomization from the placebo group to AT 100 or 200 mg (1:1 ratio)	Continuous improvements in the PPPASI and PPSI total scores through week 60 and sustained in the observational phase across all treatment groups, including the placebo-crossover groups
Anakinra (recombinant IL-1 receptor antagonist)	[140]	Phase IV DB, RPC (1:1) APRICOT study	EudraCT 2015-003600-23	64 PPP patients	100 mg/0.67 mL s.c. daily for 8 weeks	At week 8, mean difference in PPPASI -1.65 (95% CI −4.77 to 1.47) in favour of AT (but N.S.)
308-nm Excimer laser	[141]	Randomized, comparative	-	77 Chinese PPP patients	Three times weekly for 8 weeks, with different doses: low, medium or high (2-fold, 4-fold, or 6-fold of MED as initial dose, respectively)	Significant reduction of PPPASI score compared with the baseline in all groups, with a greater reduction in the high dose group
UVA1	[142]	Assessor-blinded, SA	-	62 Chinese PPP patients	Three times weekly for up to 30 sessions	At 30 sessions, PPPASI50 and PPPASI75 responses in 90.3% and 72.6% of patients, respectively
UVA1 or NB-UVB	[143]	Assessor-blinded, RC (random assignment according to a left-right randomization table)	-	66 Chinese PPP patients	Three times weekly for up to 30 sessions	At the end of the treatment period, significant improvement of the PPPASI score compared with baseline in both groups (*p* < 0.05), and mean PPPASI reduction of 6.0 (SD 2.4) in the UVA1-treated group vs. 4.4 (SD 1.4) for NB-UVB (*p* < 0.05)
FAE-PUVA or Re-PUVA	[144]	Assessor-blinded, RC (1:1)	Clinicaltrials.gov NCT00811005	21 PPP patients	Dimethylfumarate up to a 720 mg/day or acitretin 50 mg/day for 2 weeks, then addition of PUVA thrice weekly for 12 weeks or after achievement of the PPPASI90. In the maintenance 24-week phase, use of half of the last drug dose or until significant relapse, followed by another 24 weeks without any treatment	At the end of clearing phase, PPPASI90 response rates of 81.8% in the FAE-PUVA group and 90% in the Re-PUVA group (N.S.). After the maintenance phase, PPPASI90 rates of 90.9% in the FAE-PUVA arm and 70% in the Re-PUVA group (N.S.). During the follow-up period, PPPASI90 rates of 90.9% in the FAE-PUVA group and 50% in the Re-PUVA group (*p* = 0.038)
Alitretinoin	[145]	Phase 2, DB, RPC (2:1)	Clinicaltrials.gov NCT01245140	33 patients with PPP refractory to topical therapy and standard skin care	30 mg once daily for up to 24 weeks	Mean percentage change from baseline in PPPASI at week 24 (or last visit): −45.2 (SD 32.8) in the AT group vs. −44.6 (SD 45.9) in the placebo group (N.S.)
Apremilast	[146]	Phase 2, OL, SA APLANTUS study	Clinicaltrials.gov NCT04572997	21 subjects with moderate-to-severe PPP	Treatment for 20 weeks (final dose of 30 mg twice daily, gradually increased from 10 mg/day)	Median PPPASI improvement at week 20 compared to baseline of 57.1% (*p* < 0.001)
Tofacitinib	[147]	OL, SA, pilot study (primary endpoint: response of nail lesions)	ChiCTR1900025941	13 Asian patients with SAPHO syndrome accompanied by nail lesions and active PPP	5 mg, twice daily, for 12 weeks	At week 12, median improvement in PPPASI score of 71% (*p* < 0 .001)
Pamidronate disodium	[148]	OL, SA Assessment of PPP in a cohort of 30 patients with SAPHO syndrome	Clinicaltrials.gov NCT02544659 (original study in SAPHO syndrome)	25 Chinese PPP patients with SAPHO syndrome	1 mg/kg/day intravenously for 3 days at baseline and again 3 months later	PPPASI reduction > 50% in a total of 13 and 11 patients after the first and second treatment, respectively
Maxacalcitol ointment	[149]	Phase 3, DB, RPC (1:1)	-	188 Japanese patients with moderate-to-severe PPP	2 applications per day for 8 weeks	Significant decrease in the total score of skin findings in the AT group vs. placebo at week 8 or at the last visit (*p* < 0.0001)
Betamethasone butyrate propionate ointment alone or combined with maxacalcitol ointment	[150]	RC (left-right comparison)	-	29 patients with PPP (27 evaluable)	Betamethasone ointment applied once daily or betamethasone ointment + maxalcitol ointment (both applied once daily) for 8 weeks	Improvement rates in skin symptoms at week 8 significantly higher with the combination therapy than with the monotherapy

Full-text articles in English published from 1 January 2016 until 1 October 2021 were selected in the PubMed database. * For studies that recruited patients with different forms of psoriasis, only details of PP patients are reported. AT: active treatment; CGI: clinical global impression of improvement; CI: confidence interval; DB: double blind; GIS: Global Improvement Score; FAE-PUVA: fumaric acid ester + PUVA; GPP: generalized pustular psoriasis; GPPGA: Generalized Pustular Psoriasis Physician Global Assessment; GPPASI: Generalized Pustular Psoriasis Area and Severity Index; IL: interleukin; LS: least squares; mAb: monoclonal antibody; MED: minimal erythema dose; NB-UVB: narrowband ultraviolet B; N.S.: no statistically significant difference; OL: open label; PPP: palmoplantar pustulosis; PPPASI: Palmoplantar Pustulosis/Pustular Psoriasis Area and Severity Index; PPPASI50: at least 50% decrease from baseline of the PPPASI; PPPASI75: at least 75% improvement from baseline in PPPASI; PPPASI90: at least 90% reduction of the baseline PPPASI; PPSI: Palmoplantar Pustulosis Severity Index; PPPP: palmoplantar pustular psoriasis; PUVA: psoralen + ultraviolet A; RC: randomized controlled; Re-PUVA: retinoid + PUVA; RPC: randomized placebo-controlled; SA: single arm; s.c.: subcutaneously; SAPHO: synovitis, acne, pustulosis, hyperostosis, and osteitis; SD: standard deviation; TNF: tumor necrosis factor; TSS: total skin score; UVA: ultraviolet A.

**Table 4:** Clinical studies concerning treatment of PP registered at ClinicalTrials.gov database.

Status	Condition	Interventions	Phase	Participants	Study Type	Identifier Number
Recruiting	GPP	Spesolimab Placebo	2	120	RDB	NCT04399837
Recruiting	GPP	Spesolimab	2	171	OL	NCT03886246
Completed	GPP	Spesolimab Placebo	2	53	RDB	NCT03782792
Recruiting	PPP	Spesolimab	2	500	OL	NCT04493424
Completed	PPP	Spesolimab Placebo	2	152	RDB	NCT04015518
Active, not recruiting	PPP	Brodalumab Placebo	3	120	RDB	NCT04061252
Completed	GPP and various forms of psoriasis	Brodalumab	3	155	OL	NCT02052609
Completed	GPP and various forms of psoriasis	Brodalumab	4	138	OL	NCT04183881
Completed	GPP or EP	Risankizumab	3	18	OL	NCT03022045
Active, not recruiting	PPP	Risankizumab Placebo	3	116	RDB	NCT04451720
Active, not recruiting	PPP	Imsidolimab Placebo	2	59	RDB	NCT03633396
Completed	GPP	Imsidolimab	2	8	OL	NCT03619902
Completed	GPP or EP	Ixekizumab	4	12	OL	NCT03942042
Terminated	PPP or PPPP	Ustekinumab Placebo	3	33	RDB	NCT01091051
Completed	Pustular disorders, including PP	Anakinra	2	18	OL	NCT01794117
Completed	PPP	Alefacept	2	15	OL	NCT00301002
Completed	Palmoplantar psoriasis and PPPP	Infliximab	3	23	OL	NCT00686686
Recruiting	PP and various forms of psoriasis	Infliximab [infliximab biosimilar 3]	-	100	Observational	NCT03885089
Completed	Plaque psoriasis, GPP or EP	Certolizumab pegol Placebo	2/3	127	RDB	NCT03051217
Completed	PPP	Etanercept Placebo	3	15	RDB	NCT00353119
Recruiting	PPP and hidradenitis suppurativa	Recombinant anti-G-CSF receptor monoclonal antibody	1	40	OL	NCT03972280
Completed	PPP	RIST4721 Placebo	2	35	RDB	NCT03988335
Completed	PPP	Apremilast Placebo	2	90	RDB	NCT04057937

The list contains ongoing trials or completed trials not included in Table 3 or with unpublished results (website accessed on 1 October 2021). EP: erythrodermic psoriasis; G-CSF: granulocyte colony-stimulating factor; GPP: generalized pustular psoriasis; OL: open-label study; PP: pustular psoriasis; PPP: palmoplantar pustulosis; PPPP: palmoplantar pustular psoriasis; RDB: randomized, double-blind study.

## Data Availability

No new data were created or analyzed in this study. Data sharing is not applicable to this article.
